# Author Correction: Experimental evidence for the existence of a second partially-ordered phase of ice VI

**DOI:** 10.1038/s41467-021-22085-4

**Published:** 2021-03-15

**Authors:** Ryo Yamane, Kazuki Komatsu, Jun Gouchi, Yoshiya Uwatoko, Shinichi Machida, Takanori Hattori, Hayate Ito, Hiroyuki Kagi

**Affiliations:** 1 Geochemical Research Center, Graduate School of Science, The University of Tokyo, 7-3-1 Hongo, Bunkyo-ku, Tokyo, Japan; 2 The Institute for Solid State Physics, The University of Tokyo, 5-1-5 Kashiwanoha, Kashiwa, Chiba Japan; 3 Neutron Science and Technology Center, CROSS, 162-1 Shirakata, Tokai, Naka, Ibaraki, Japan; 4 J-PARC Center, Japan Atomic Energy Agency, 2-4 Shirakata, Tokai, Naka, Ibaraki, Japan

**Keywords:** Chemical physics, Phase transitions and critical phenomena, Structure of solids and liquids

Correction to: *Nature Communications* 10.1038/s41467-021-21351-9, published online 18 February 2021.

The original version of the Supplementary information associated with this Article contained errors in Supplementary Table 1. The correct version of the 17th row of the 2nd column states ‘0.211(2)’ instead of the original, incorrect ‘0.221(4)’; the correct version of the 14th row of the 3rd column states ‘0.954(2)’ instead of the original, incorrect ‘0.954(4)’; the correct version of the 19th row of the 5th column states ‘0.43(3)’ instead of the original, incorrect ‘0.45(3)’.

The original version of the Supplementary information associated with this Article also contained an error in Supplementary Table 2. The correct version of the 11th row of the 4th column states ‘0.43(3)’ instead of the original, incorrect ‘0.45(3)’.

The original version of the Supplementary information associated with this Article also contained errors in Supplementary Table 3. The correct version of the 14th row of the 2nd column states ‘0.705(3)’ instead of the original, incorrect ‘0.468(2)’; the correct version of the 14th row of the 3rd column states ‘0.684(3)’ instead of the original, incorrect ‘0.971(3)’; the correct version of the 14th row of the 4th column states ‘0.134(5)’ instead of the original, incorrect ‘0.130(6)’; the correct version of the 15th row of the 3rd column states ‘0.971(3)’ instead of the original, incorrect ‘0.035(2)’.

The HTML has been updated to include a corrected version of the Supplementary information.

## Supplementary information

Supplementary Information

