# Supplementary material for: Experimental evidence for the existence of a second partially-ordered phase of ice VI
Source: Nat Commun. 2021 Feb 18;12:1129. doi: 10.1038/s41467-021-21351-9 (PMC7893076; doi:10.1038/s41467-021-21351-9)
Supplement: Supplementary file 1 — Supplementary Information [file 41467_2021_21351_MOESM1_ESM.pdf]

Experimental evidence for the existence of a second partially-ordered phase of ice VI

Ryo Yamane, Kazuki Komatsu, Jun Gouchi, Yoshiya Uwatoko, Shinich Machida, Takanori Hattori, Hayate Ito, and Hiroyuki Kagi

Correspondence to: r.yamane@issp.u-tokyo.ac.jp

Contents

Supplementary Note

1 Dielectric measurements

2 Neutron diffraction measurements

3 Measured values for the dielectric properties of ice VI and its hydrogen-ordered phases

Supplementary Method

1 High-pressure cell for dielectric measurement

2 Determination of phase transition temperature of ice VI and its hydrogen-ordered phases

3 Details of the 18 candidates

4 "Lower symmetry" space group

Supplementary References

Supplementary Note 1 Dielectric measurements

Supplementary Figure 1 shows the hysteresis of the phase transition temperature between ice VI and XIX at 1.9 GPa.

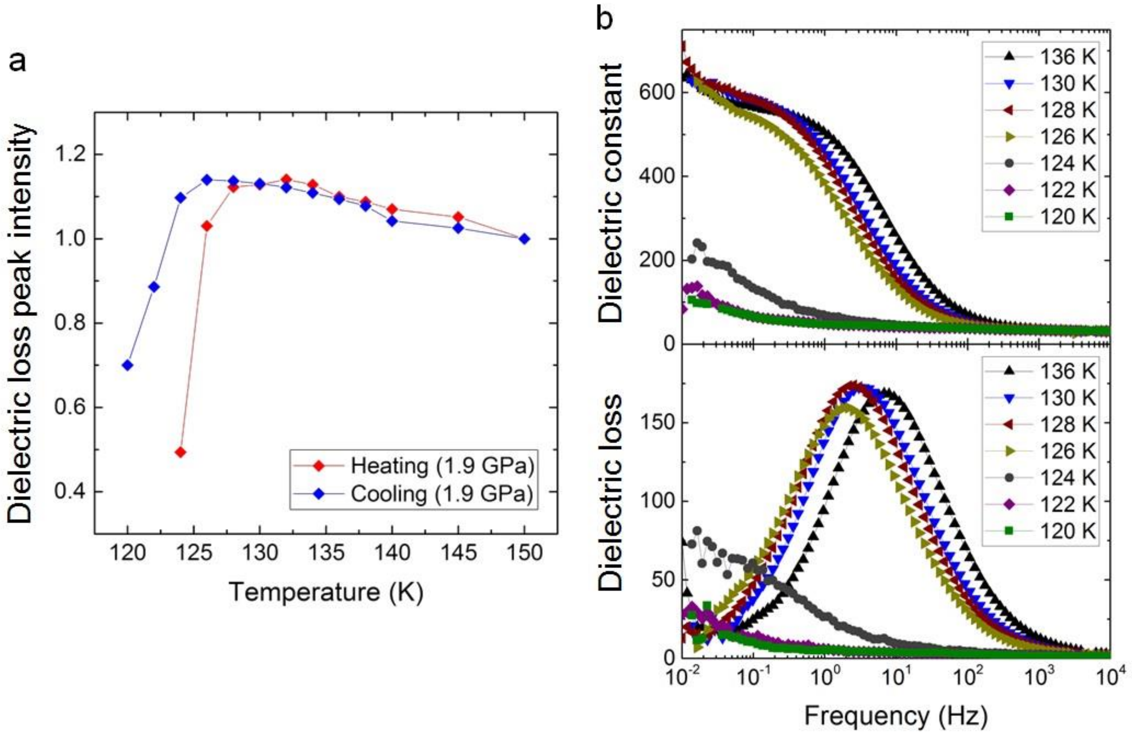

Supplementary Figure 1| Comparison of the phase transition in cooling and heating runs at 1.9 GPa. **a,** Each peak intensity of dielectric loss is normalised by that obtained at the highest temperature in each run. At temperatures lower than the lowest temperature points shown in the figure, the dielectric response of ice XIX almost disappeared in the measured frequency region; thus, the peak intensity in that region is not shown here. Each of the cooling and heating results corresponds to the data shown in Fig. 2a (main text) and this Supplementary Fig. 1b, respectively. **b,** Dielectric constant and dielectric loss of HCl-doped ice VI and its hydrogen-ordered phase (ice XIX) obtained at 1.9 GPa upon heating. The measured frequency ranged from 3 mHz to 2 MHz.

Supplementary Figure 2 shows temperature dependence of dielectric loss peak intensity of DCl-doped D_2O ice VI and its deuterium ordered phases obtained from 1.1 to 2.0 GPa. Although the data are relatively dispersed compared to that of the H_2O, hydrogen-ordering of ice VI is also observed in the D_2O samples. The phase transition temperature at 1.6 GPa (119 K) is consistent with that observed in the neutron diffraction measurements (~118 K in Fig. 3b). As also observed in the neutron diffraction measurements, the positive slope of d*T*/d*P* can be confirmed from the transition temperatures above 1.6 GPa.

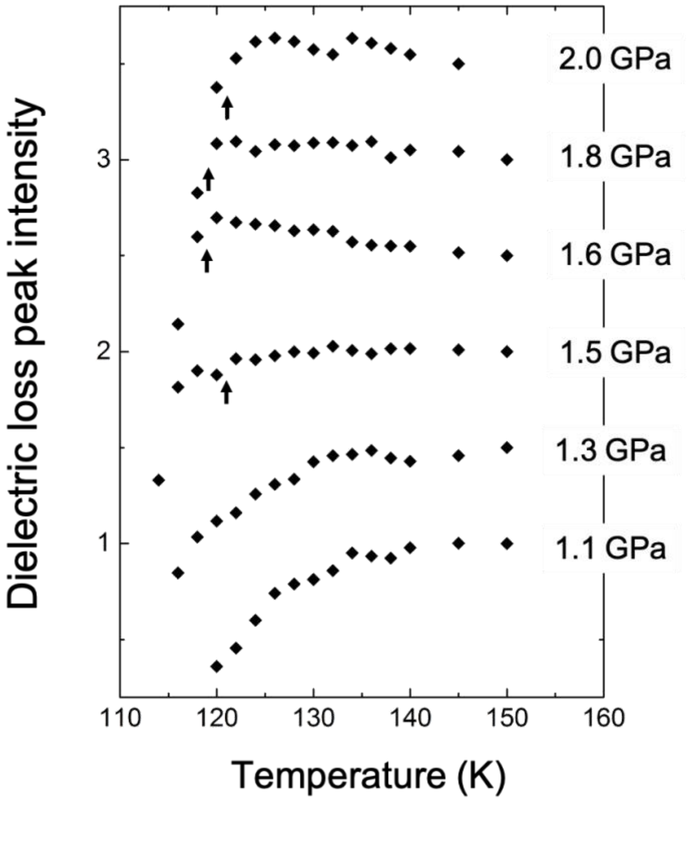

Supplementary Figure 2| Temperature dependence of dielectric loss peak intensity of DCl-doped D_2O ice VI and its deuterium ordered phases obtained from 1.1 to 2.0 GPa. Peak intensities are normalised by that obtained at the highest temperature in each pressure run. Each plot was shifted by 0.5 with increasing measured pressure for clarity. The transition temperatures are indicated by

black arrows except for 1.1 and 1.3 GPa, whose data exhibit ambiguous change along with the hydrogen ordering. Detailed procedures for the transition temperatures are described later.

Temperature dependence of relaxation time of HCl-doped ice VI and DCl-doped deuterated ice VI is shown in Supplementary Fig. 3. The activation energy of HCl-doped ice VI and DCl-doped deuterated ice VI is about 0.2 eV, which is consistent with those of other HCl/DCl-doped and also KOH/KOD-doped disordered ice, such as ice V[1] and I_h[2]. On the other hand, the phase transition between ice VI/XV and DCl-doped ice VI/XIX show a relatively small change in their relaxation times. The previous dielectric study on ice V and Ih reported a similar isotope effect in their hydrogen (heavy hydrogen) ordering. Although the reason for this difference is not yet clear, Kawada (1989) indicated that the degree of hydrogen/heavy hydrogen ordering would be related to the difference[1]. As with the isotope effect, the difference in the degree of hydrogen-ordering might also be the reason for the relatively small change in the relaxation time of the phase transition from ice VI to ice XV compared to that between ice VI and XIX. Figure 3-2 shows the temperature dependence of dielectric loss peak intensity obtained in cooling and heating runs at 0.88 and 2.2 GPa, where ice XV and XIX are stable, respectively. It can be seen that the hydrogen ordering of ice XV happens in a wider temperature region (~20 K) compared to that of ice XIX (~5 K). These results are consistent with the supposed difference in the degree of hydrogen-ordering between ice XV and XIX. The difference would be due in part to the height of the activation barrier of hydrogen ordering and energy difference between ice VI and the hydrogen-ordered phases. However, we need further investigation for such discussion.

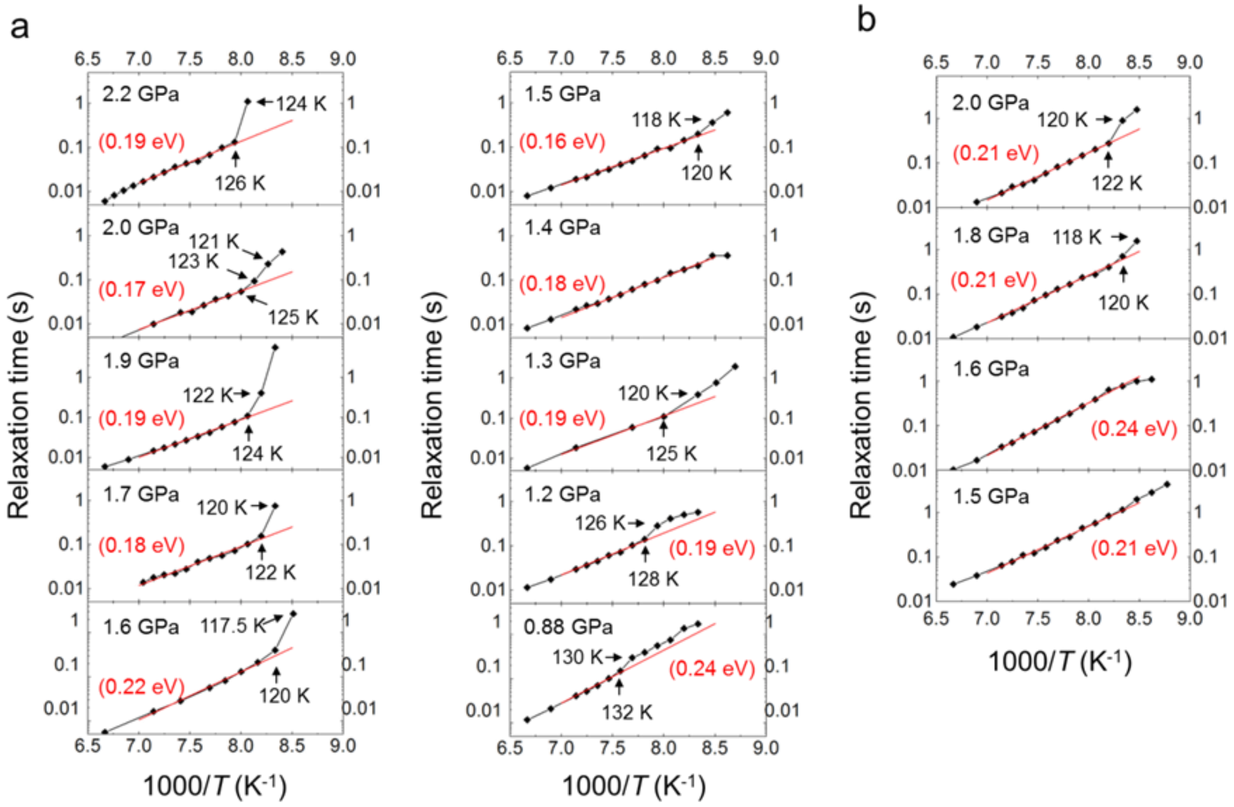

Supplementary Figure 3| Temperature dependence of relaxation time of HCl-doped ice VI (a) and DCl-doped deuterated ice VI (b). The red lines were fitted based on the Arrhenius equation and their activation energies are also shown.

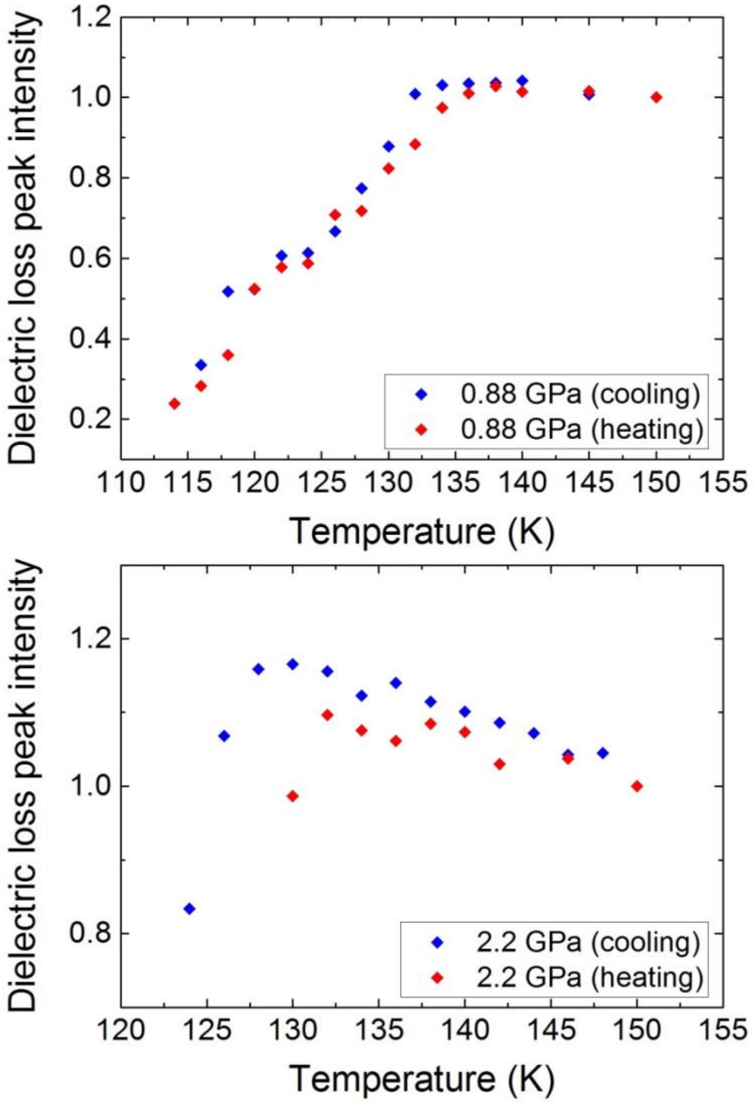

Supplementary Figure 4| Temperature dependence of dielectric loss peak intensity obtained in cooling and heating runs at 0.88 and 2.2 GPa, where ice XV and XIX is stable, respectively. At 2.2 GPa, data measured below 124 K (cooling run) and 130 K (heating run) are not shown, because the dielectric response of ice XIX almost disappeared in the measured frequency region.

Supplementary Note 2 Neutron diffraction measurements

Supplementary Figure 5 shows a comparison of neutron diffraction patterns obtained at 1.6 and 2.2 GPa. The diffraction pattern at the 2.2 GPa shows obvious change along with the hydrogen ordering as with the case of 1.6 GPa. The important new two peaks at around 2.2 Å (indicated by blue ticks), which is the evidence that ice XV and XIX have distinct crystal structures, are also observed. It should be mentioned that ice VIII (indicated by a black tick) coexisted under the 2.2 GPa. Ice VIII already appeared from 150 K and its existence would not affect the Gibbs's energy of ice VI and XIX (in other words, their transition temperature).

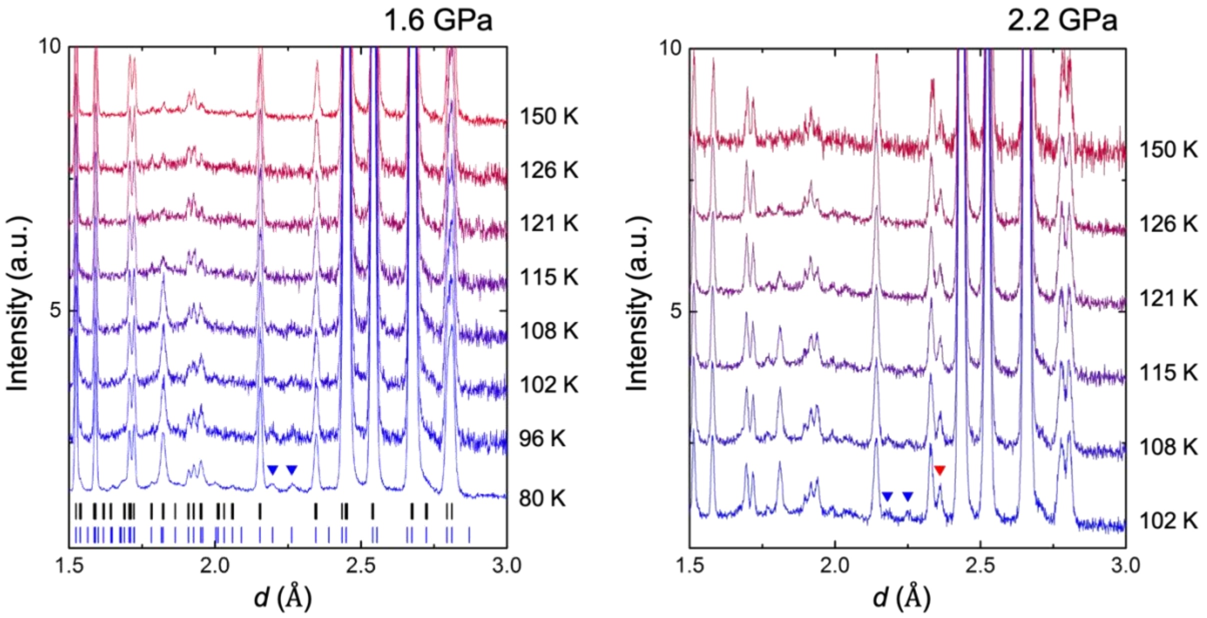

Supplementary Figure 5| Comparison of neutron diffraction patterns obtained at 1.6 and 2.2 GPa. Blue ticks indicate new peaks at around 2.2 Å, which do not appear from the unit cell of ice XV. The peak indicated by the red tick is derived from ice VIII.

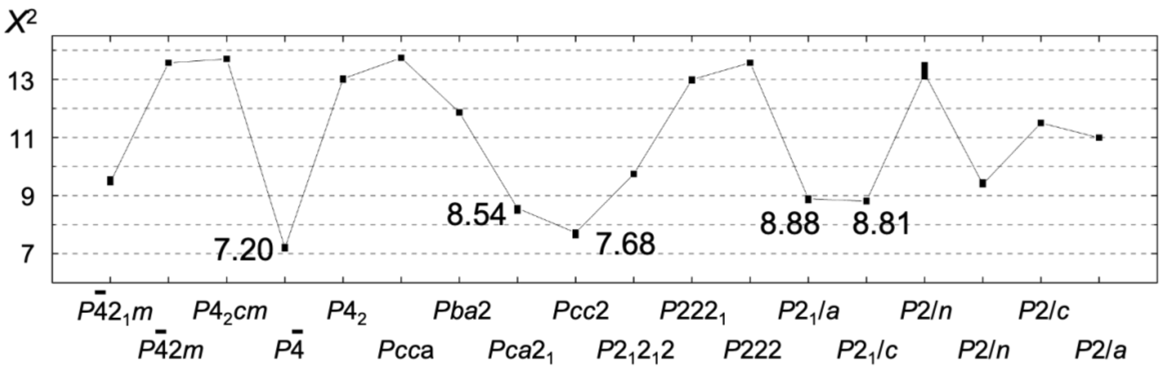

Supplementary Figure 6| Comparison of χ^2 values obtained by Rietveld analysis for the neutron diffraction pattern obtained at 1.6 GPa and 80 K using the 18 structure models. In the first step of structure refinements, the site occupancies of hydrogen atoms were fitted one by one, and subsequently fitted together as variables. In the first step of structure refinements, the site occupancies of hydrogen atoms were fitted one by one, and subsequently fitted together as variables. Since the first step has arbitrariness in its fitting order (e.g. $\alpha \to \beta \to \cdots$ and $\beta \to \alpha \to \cdots$), we conducted structure refinements in several ways for each model by changing the fitting order cyclically, such as $\alpha \to \beta \to \cdots$ and $\beta \to \gamma \to \cdots$. However, as shown in the figure, the fitting results are almost independent of the order. The numerical values are shown only for five candidates with $\chi^2 < 9$, which are averaged values over several refinement results. Site occupancies of hydrogen atoms were initially refined by fixing the atomic positions of hydrogen.

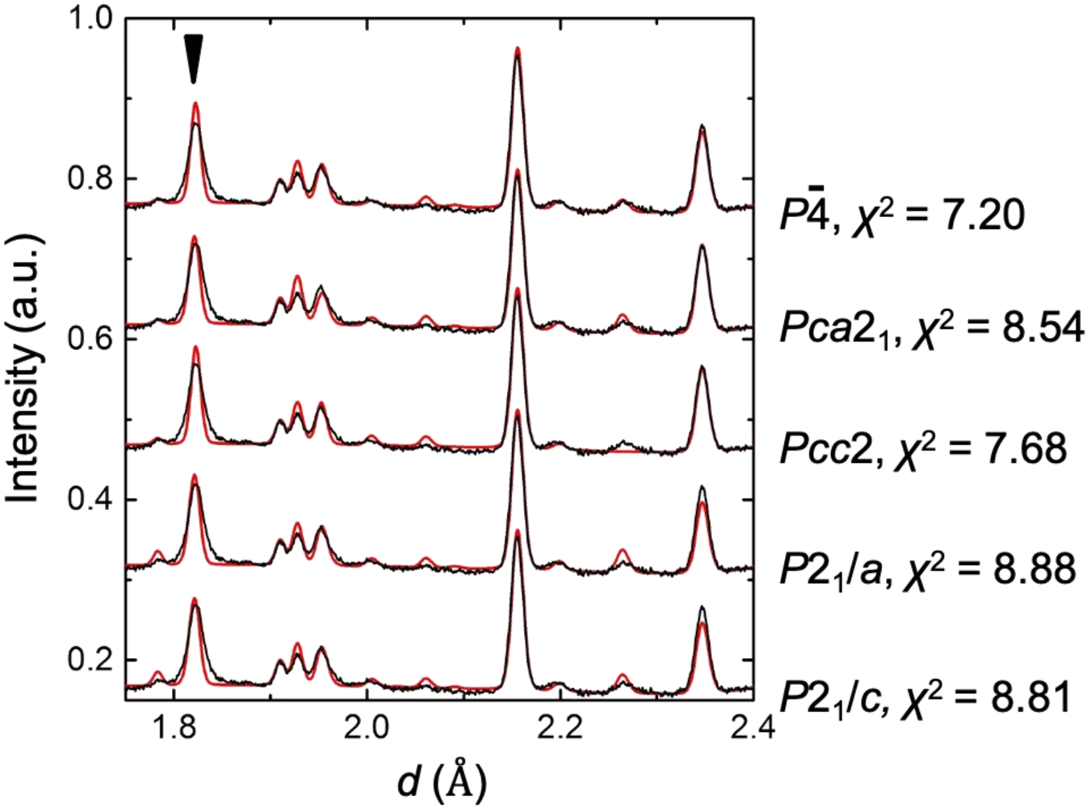

Supplementary Figure 7| Comparison of structure refinements of the five possible candidates. Black lines show the neutron diffraction patterns observed at 1.6 GPa and 80 K. The results of the simulation for each model are shown by red lines. Space groups and χ^2 values of the models are listed on the right-hand side of this figure. The black triangle indicates a Bragg peak that shows peak broadening compared to the simulated results.

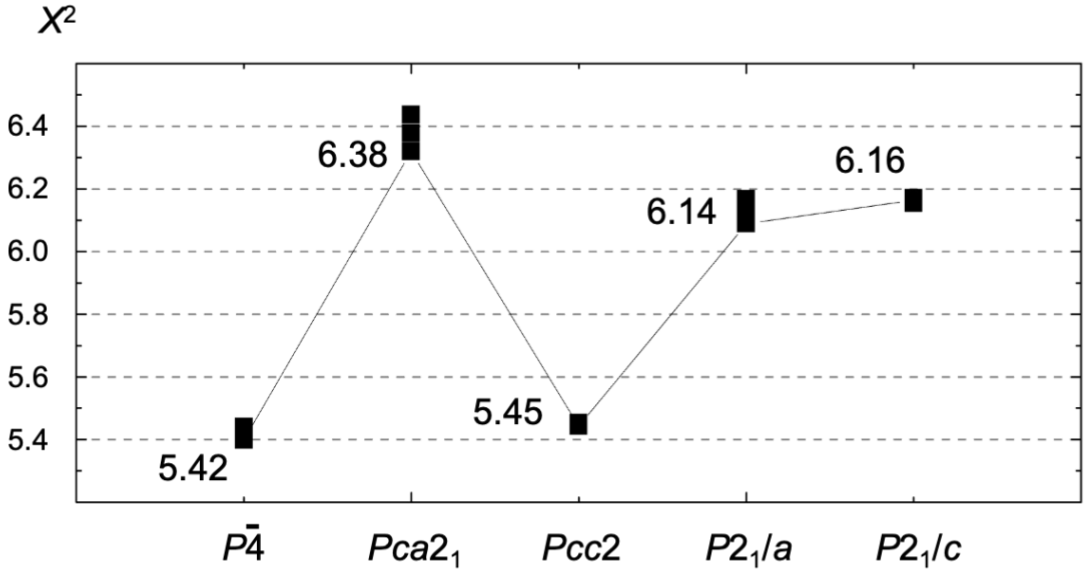

Supplementary Figure 8| Comparison of χ^2 values obtained by Rietveld analysis for neutron diffraction pattern at 1.6 GPa and 80 K using the five plausible structure models. In the refinements, atomic positions of hydrogen as well as the site occupancies of hydrogen atoms are refined. The χ^2 values shown for each structure model is the averaged value obtained over repeated refinements.

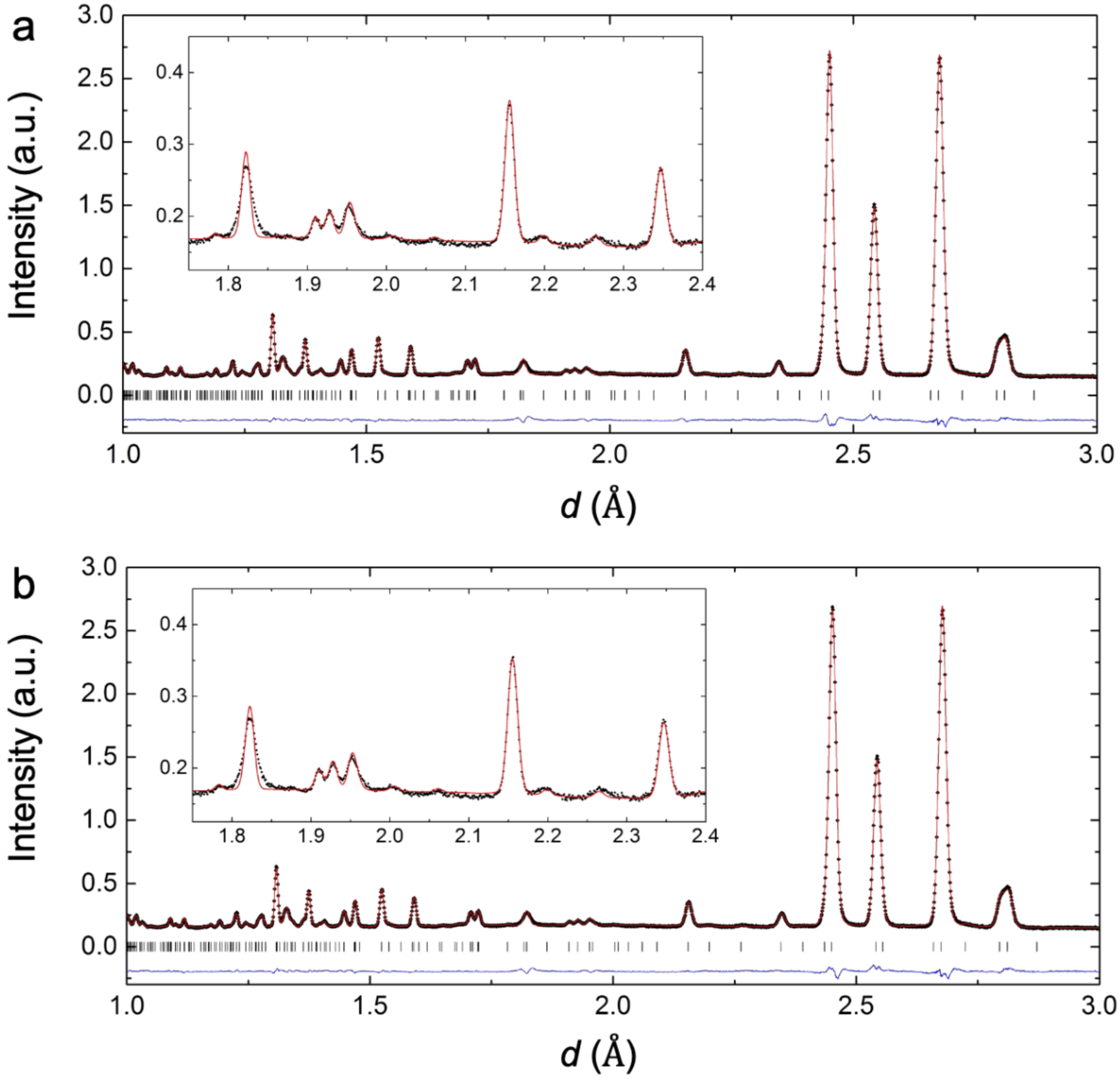

Supplementary Figure 9| Neutron diffraction patterns collected at 1.6 GPa and 80 K (black dots) and finally fitted lines (coloured by red) using the most plausible structure models for ice XIX, $P\bar{4}$ (a) and *Pcc*2 (b). The black ticks represent all the peak positions expected from the unit cells of ice XIX. The blue lines show residuals between the observed and simulated diffraction patterns.

Supplementary Table 1| Atomic fractional coordinates for ice XIX using $P\bar{4}$ (tetragonal) structure model (lattice parameters: $a = b = 8.61942(7)$ and $c = 5.59301(8)$)

Atom	x	y	z	Occupancy
O1a	0.0	0.5	-0.009(3)	1.0
O1b	0.0	0.0	0.5	1.0
O1c	0.5	0.5	0.5	1.0
O2a	0.1447(16)	0.6355(14)	0.383(2)	1.0
O2b	0.3553(15)	0.8666(16)	0.3900(19)	1.0
O2c	0.8637(15)	0.8617(12)	0.126(2)	1.0
O2d	0.6506(11)	0.6415(12)	0.130(2)	1.0
D1a	0.116(5)	0.610(5)	0.225(3)	0.312(10)
D1b	0.897(3)	0.895(3)	0.279(3)	0.5
D1c	0.395(3)	0.898(3)	0.237(2)	0.688(10)
D1d	0.5823(18)	0.607(2)	0.250(3)	0.5
D2a	0.064(2)	0.557(3)	0.092(2)	0.688(10)
D2b	0.942(2)	0.954(2)	0.376(2)	0.5
D2c	0.433(4)	0.945(6)	0.112(5)	0.312(10)
D2d	0.555(3)	0.570(2)	0.402(3)	0.5
D3a	0.211(2)	0.723(2)	0.375(4)	0.57 (3)
D3b	0.785(3)	0.785(3)	0.123(6)	0.5
D3c	0.283(4)	0.784(3)	0.377(6)	0.43(3)
D3d	0.721(3)	0.726(2)	0.130(5)	0.5
D4a	0.831(2)	0.9524(19)	0.039(4)	0.666(2)
D4b	0.163(4)	0.544(3)	0.473(6)	0.34(2)
D4c	0.665(3)	0.5526(19)	0.030(4)	0.67(2)
D4d	0.386(4)	0.956(3)	0.479(6)	0.22(6)
D4e	0.9476(19)	0.336(2)	0.467(3)	0.78(6)
D4f	0.560(2)	0.695(3)	0.083(5)	0.33(2)
D4g	0.550(2)	0.168(3)	0.465(4)	0.66(2)
D4h	0.953(3)	0.823(4)	0.047(7)	0.33(2)

Supplementary Table 2| O-D bond lengths of ice XIX using $P\bar{4}$ (tetragonal) structure model

Oxygen atom	Deuterium atom	Bond length	D site occupancy
O1a	D2a	0.93(3)	0.688(10)
	D2c	0.94(4)	0.312(10)
O1b	D2b	0.942(15)	0.5
O1c	D2d	0.94(2)	0.5
O2a	D1a	0.94(3)	0.312(10)
	D3a	0.95(3)	0.57(3)
	D4b	0.95(4)	0.34(2)
	D4e	0.96(3)	0.78(6)
O2b	D1c	0.96(2)	0.688(10)
	D3c	0.95(4)	0.43(3)
	D4d	0.95(4)	0.22(6)
	D4g	0.96(3)	0.66(2)
O2c	D1b	0.95(3)	0.5
	D3b	0.95(3)	0.5
	D4a	0.96(3)	0.666(2)
	D4h	0.95(4)	0.33(2)
O2d	D1d	0.94(2)	0.5
	D3d	0.95(3)	0.5
	D4c	0.96(3)	0.67(2)
	D4f	0.94(3)	0.33(2)

Supplementary Table 3| Atomic fractional coordinates for ice XIX using *Pcc*2 (orthorhombic) structure model (lattice parameters: $a = 8.6278(4)$, $b = 8.6114(4)$, and $c = 5.59299(8)$)

Atom	x	y	z	Occupancy
O1	0.2558(15)	0.749(2)	0.757(4)	1.0
O2a	0.3886(15)	0.8943(15)	0.1385	1.0
O2b	0.1105(14)	0.1072(14)	0.888(2)	1.0
O2c	0.1082 (13)	0.3964(15)	0.640(4)	1.0
O2d	0.6120(12)	0.6163(12)	0.873(3)	1.0
D1a	0.334 (3)	0.858(4)	0.002(4)	0.43(2)
D1b	0.159(3)	0.148(4)	0.025(5)	0.45(2)
D1c	0.142(3)	0.357(4)	0.492(5)	0.520(19)
D1d	0.629(2)	0.649(3)	0.032(3)	0.60(6)
D2a	0.314(3)	0.813(2)	0.858(4)	0.57(2)
D2b	0.188(2)	0.206(2)	0.143(4)	0.55(2)
D2c	0.194(3)	0.322(2)	0.343(4)	0.480(19)
D2d	0.705(3)	0.684(3)	0.134(5)	0.40(6)
D3a	0.468(2)	0.971(3)	0.130(6)	0.5
D3b	0.027(2)	0.035(2)	0.889(5)	0.5
D3c	0.026(2)	0.468(2)	0.643(5)	0.5
D3d	0.5403(19)	0.5313(18)	0.885(5)	0.5
D4a	0.058(3)	0.194(3)	0.823(6)	0.340(11)
D4b	0.4233(19)	0.8053(19)	0.225(4)	0.78(6)
D4c	0.8059(16)	0.0843(19)	0.281(4)	0.71(3)
D4d	0.202(2)	0.415(5)	0.726(6)	0.32(3)
D4e	0.634(3)	0.710(3)	0.789(7)	0.22(6)
D4f	0.913(2)	0.300(2)	0.218(5)	0.660(11)
D4g	0.2908(15)	0.588(2)	0.300(4)	0.68(3)
D4h	0.707(3)	0.931(5)	0.702(7)	0.29(3)

Supplementary Table 4| O-D bond lengths of ice XIX using *Pcc2* (orthorhombic) structure model

Oxygen atom	Deuterium atom	Bond length	D site occupancy
O1a	D2a	0.94(3)	0.57(2)
	D2b	0.95(3)	0.55(2)
	D2c	0.94(3)	0.48(19)
	D2d	0.95(4)	0.40(6)
O2a	D1a	0.95(3)	0.43(2)
	D3a	0.95(3)	0.5
	D4b	0.95(3)	0.78(6)
	D4h	0.95(4)	0.29(3)
O2b	D1b	0.94(4)	0.45(2)
	D3b	0.95(3)	0.5
	D4a	0.95(3)	0.340(11)
	D4c	0.96(3)	0.71(3)
O2c	D1c	0.94(4)	0.520(19)
	D3c	0.94(3)	0.5
	D4d	0.95(3)	0.32(3)
	D4f	0.96(3)	0.660(19)
O2d	D1d	0.94(3)	0.60(6)
	D3d	0.96(2)	0.5
	D4e	0.95(4)	0.22(6)
	D4g	0.96(2)	0.68(3)

Supplementary Note 3 Measured values for the dielectric properties of ice VI and its hydrogen-ordered phases

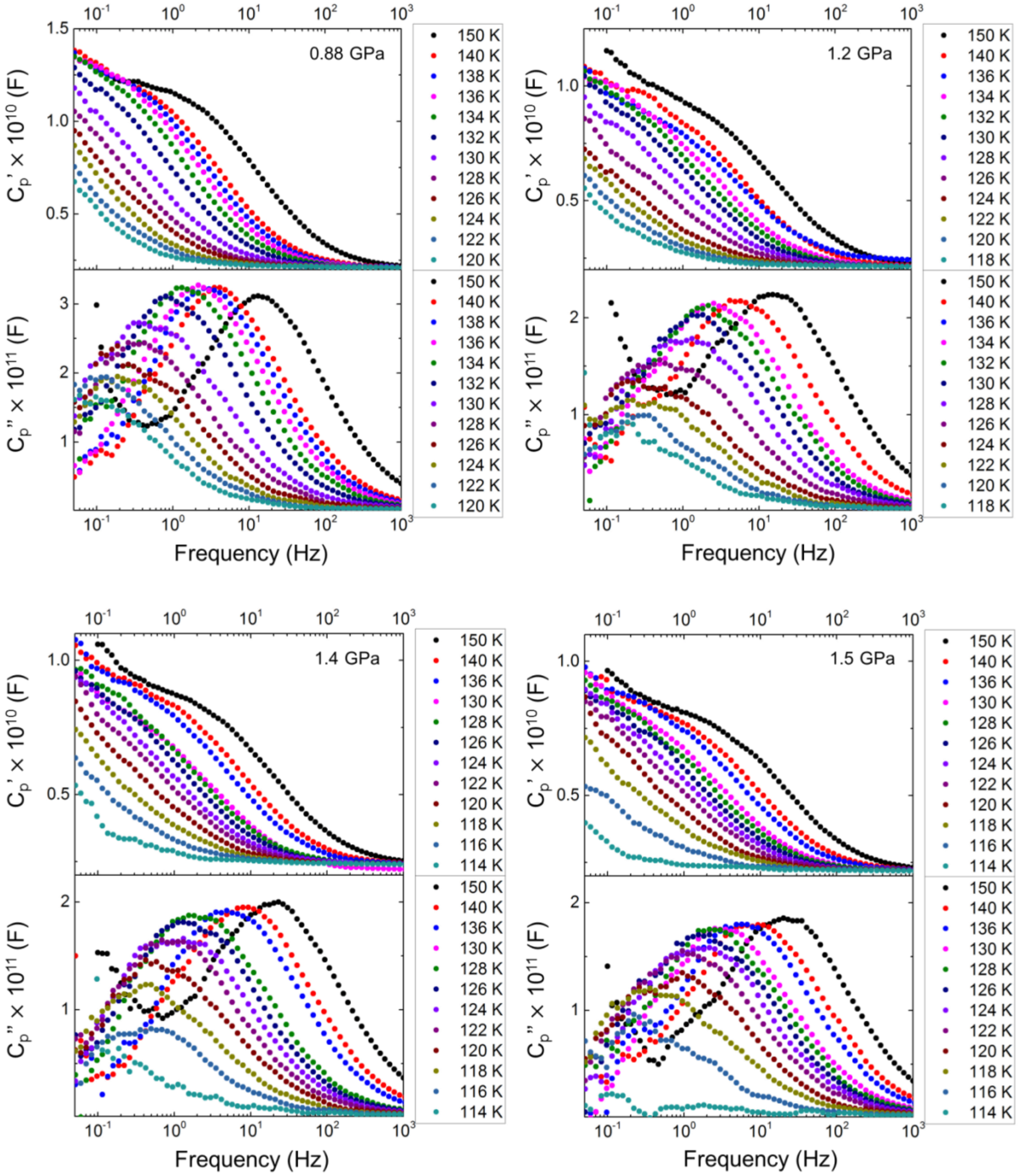

Supplementary Figure 10| Measured complex capacitance (C_p’ and C_p’’) of ice VI and its hydrogen-ordered phases

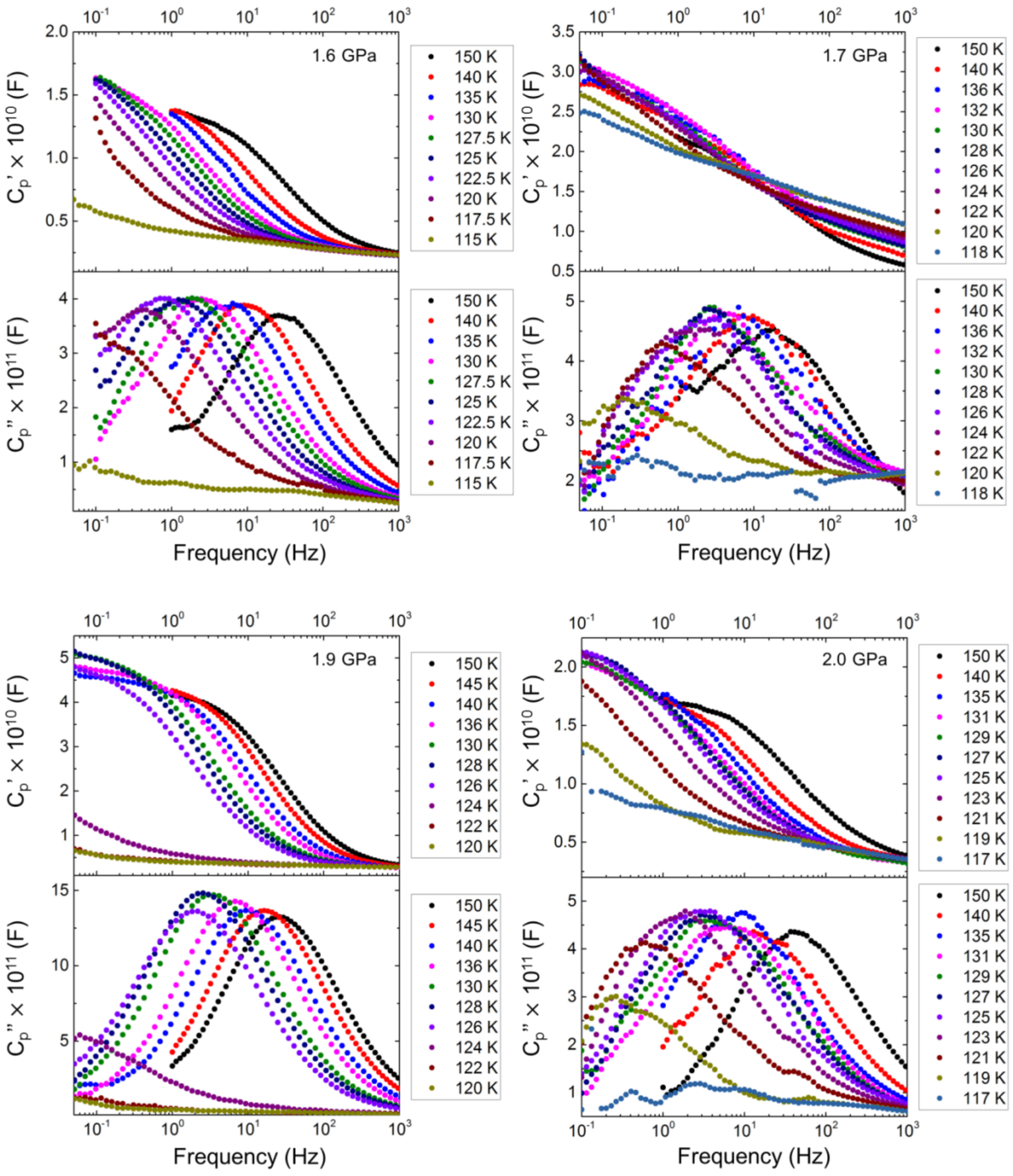

Supplementary Figure 10 (continuous)| Measured complex capacitance (C_p’ and C_p’’) of ice VI and its hydrogen-ordered phases. It is noted, particularly in 1.7 GPa and 2.2 GPa (shown in the following continuous figure), stray capacity significantly appears in the C_p’ data as background.

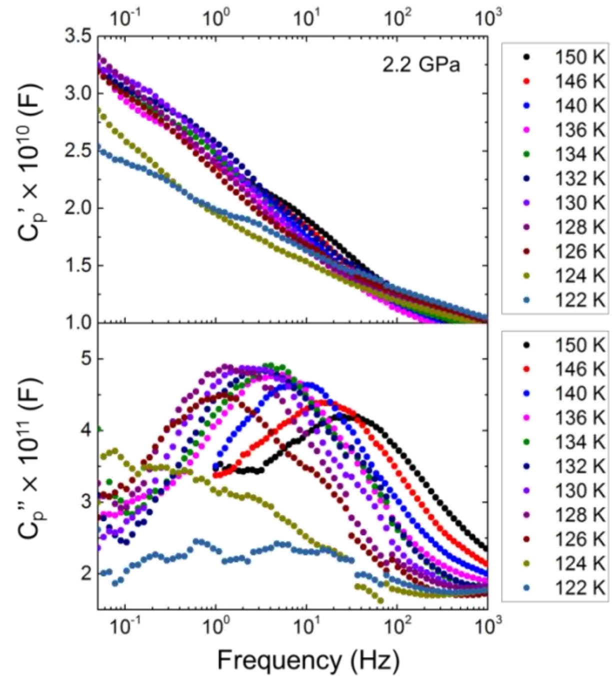

Supplementary Figure 10| Measured complex capacitance (C_p' and C_p'') of ice VI and its hydrogen-ordered phases

Supplementary Method 1 High-pressure cell for dielectric measurement

Dielectric experiments were conducted using a newly developed cell assembly for in-situ dielectric measurements under high pressure (Supplementary Fig. 11). The cell assembly is based on a piston-cylinder type high-pressure apparatus. The left side of Supplementary Fig. 11 shows an overall of the piston-cylinder cell. The Developed cell assembly is shown on the right side of Supplementary Fig. 11. A sample is loaded along to the vertical direction, and electric leads are introduced into the sample holder, PTFE capsule, through the holed CuBe plug. Plastic fiber (Edmund Optics, Φ 0.25 mm) is introduced together for the in-situ pressure calibration using ruby fluorescence methods. The sample is sealed by epoxy resin (STYCAST 2850) immersed with the Cu leads and plastic fiber. If the volume of the epoxy resin is too small compared with the plastic fiber and leads, the epoxy resin cannot keep sample pressure under compression; this means that the sample blows out through the hole. For a similar reason, it is necessary to put epoxy resin on the plug as high as 1.8 mm from the top side of the CuBe plug (this value has been optimized). A small ruby tip (almost the same size of the diameter of plastic fiber) is introduced below the electrode and exposed from the incident 532 nm laser beam travel through the plastic fiber, and the induced fluorescence was also traveled through the fiber to the detector (Ocean optics, USB2000+). The parallel electrodes are fixed on the epoxy resin in the cell assembly. The vertically fixed electrodes ensure a condition that electrode separation and area are constant under compression. It should be noted that the length of the electrode (about 6.0 mm) is an important parameter not to collapse during the compression. If we lengthen the electrodes to expand electrode area (corresponding to enlarge sample capacitance from a principle equation, $C = \varepsilon_0 \varepsilon \, S/d$), electrode deformation might occur. It may also cause short-circuit between the electrodes. From our experience, the electrode length must be less than 7.0 mm at most, which is the half-length of

the initial sample space. The cell assembly allows us to measure dielectric properties of the liquid sample under high pressure.

Supplementary Fig. 12 shows an example of dielectric data of non-doped ice VI obtained at 1.73 GPa and 240 K. Supplementary Fig. 12a and b show temperature dependence of the dielectric constant and loss of non-doped ice VI obtained at 1.73 GPa, respectively. Similar temperature dependence of dielectric properties of non-doped ice VI was reported by Johari *et al*. at 1.1 GPa[3]. The dielectric response in the lower frequency region (below 10 Hz at 240 K) is derived from the DC electric conductivity of the sample. The temperature dependence of dielectric constants and loss shows that the frequency dispersion derived from the molecular rotation (10^3 Hz at 240 K) shifts to a lower frequency with decreasing temperature. This means that the dynamics of molecular rotation becomes slowdown with decreasing temperature. As the most important point shown in Figure S7, the dielectric response of non-doped ice VI almost disappears at 160 K in the measured frequency range due to the slowdown of the molecular-rotation dynamics. No dielectric response was shown at the hydrogen-ordering temperature of ice VI observed in HCl-doped sample (at ~120 K). The reason for the different dielectric responses between the pure and HCl-doped ices is that the chemical dopant locally breaks the ice rules by which the molecular reorientation can be activated. This activation causes clear differences in terms of the dielectric responsibility of ice. Quantitatively, pure ice VI has higher activation energy for the molecular reorientation, ~0.5 eV (measured at 1.1 GPa in Johari et al.[3]), compared to that of the HCl-doped ice VI, ~0.2 eV (Supplementary Fig. 3).

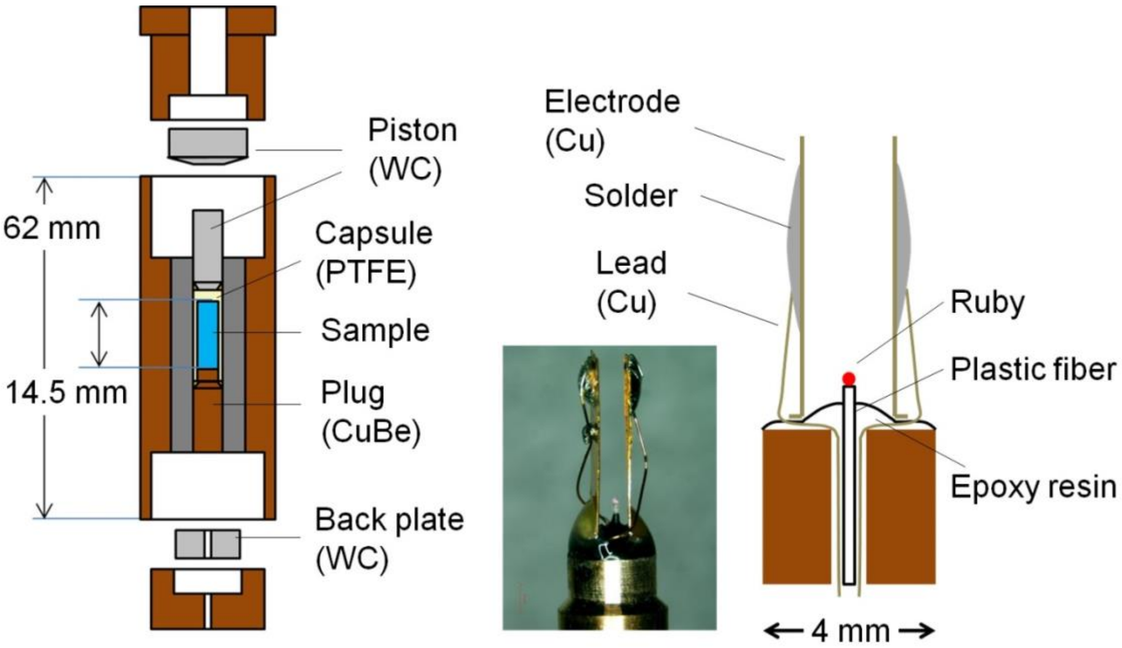

Supplementary Figure 11| Developed cell assembly of piston cylinder for in-situ dielectric measurements under high pressure. An overall drawing (left) and a picture (center) and schematic drawing around sample space (right) of the developed cell assembly. Separated pistons are made of tungsten carbide abbreviated WC in the figure and cylinder is made of CuBe (outer, colored blown) and NiCrAl (inner, colored gray). The two electrodes compose parallel electrodes.

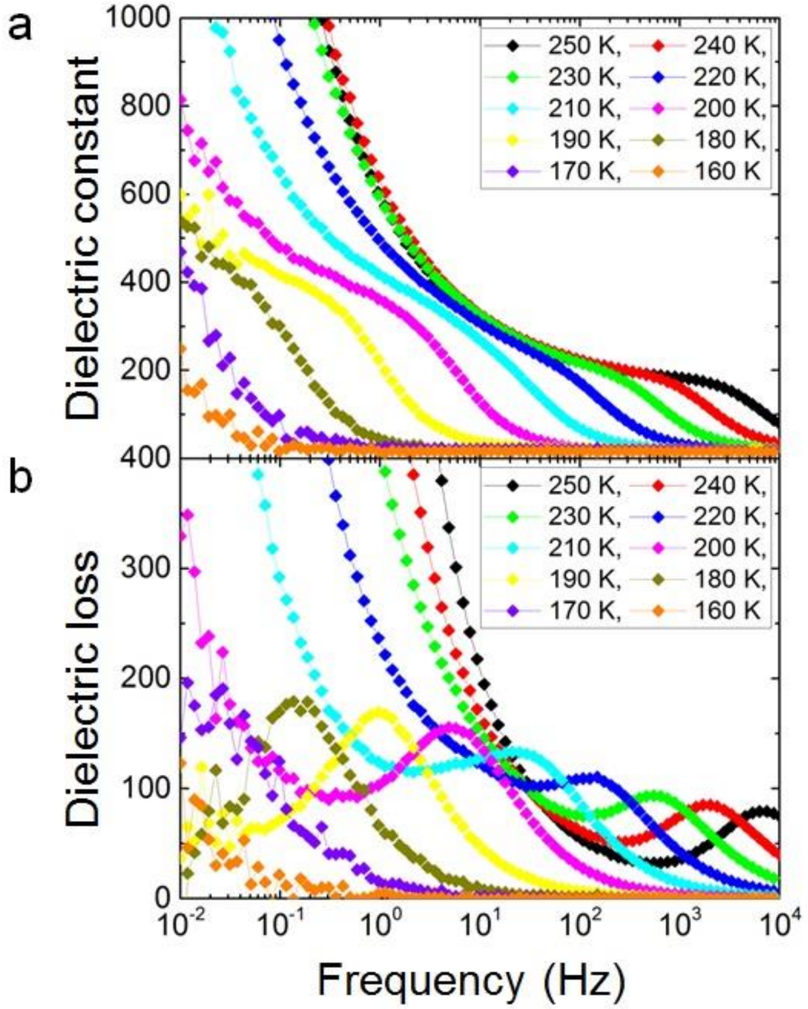

Supplementary Figure 12| Dielectric properties of non-doped ice VI. **a** and **b,** Temperature dependence of dielectric constant and loss of non-doped ice VI obtained at 1.73 GPa.

Supplementary Method 2 Determination of phase transition temperature of ice VI and its hydrogen-ordered phases

In the determination of phase transition from ice VI and its hydrogen-ordered phases, the DLPI of ice VI is assumed to be linearly dependent on temperature, and the phase transition temperature is redefined as the temperature at which the DLPI starts to deviate from the linearity. This deviation is caused by the hydrogen-ordering of ice VI as mentioned in our original manuscript. The following is the procedure for determining the deviation, taking the case of 1.9 GPa as an example. The raw DLPI data in the cooling run at 1.9 GPa are shown below (Supplementary Fig. 13).

1. First, it is obvious that the hydrogen ordering transitions occurred above 122 K in the cooling run. From this temperature, the DLPI data were obtained at several temperature points. In the case of the cooling run, {122, 124, 126, 128, 130, and 132} were selected. Hereafter, the number of selected temperature points is denoted by *N*.

2. Let us consider a temperature set selected in the same manner of step 1. We represent an element of the temperature set by T_i, where *i* takes from 1 to *N* and $T_i < T_{i+1}$. Linear fitting is conducted for the DLPI data, $\{d_i, \cdots, d_{\max}\}$, obtained in the temperature range from T_i to the highest temperature of the measurement ($T_{\max}$; $T_{\max}$ was 150 K at 1.9 GPa). The fitted DLPI data are denoted by $\{\hat{d}_i, \cdots, \hat{d}_{\max}\}$.

3. We calculate the residual sum of squares (RSS) between the observed $\{d_i, \cdots, d_{\max}\}$ and the fitted $\{\hat{d}_i, \cdots, \hat{d}_{\max}\}$, and then the RSS is normalized by the number of the DLPI data, $\{d_i, \cdots, d_{\max}\}$. Hereafter, the normalized RSS is denoted by R_i. Let us consider that we decrease *i* from *N* (corresponding to a decrease of temperature T_i). When the hydrogen ordering happens, the normalized RSS should become large compared to that obtained above transition temperature due

to the deviation from the linearity. This behavior can be shown in the following figure at around 126 K in the cooling run (Supplementary Fig. 14).

4. Finally, to judge the phase transition temperature, we evaluate R_i/R_{i+1} (i takes from 1 to N-1). The figure below shows the temperature dependence of the ratio R_i/R_{i+1} obtained at various pressures (Supplementary Fig. 15). In this figure, the values of the ratio are nearly 1 in the higher temperature region where ice VI is stable. This is because fitting residuals of R_i and R_{i+1} take similar values owing to good linearity between DLPI and measured temperature.

Based on the results, Supplementary Fig. 16 shows the phase diagram of ice VI and its hydrogen-ordered phases, determined using the ratio R_i/R_{i+1} of 1.5, 2, and 3 as criteria values for the phase transition (Supplementary Fig. 16). The transition temperature is determined by (T_i + T_{i+1})/2, whose R_i/R_{i+1} is first above the criteria with decreasing i of T_i from N. The displayed error bars show the temperature range from T_i to T_{i+1}. The three phase diagrams are only slightly different, and the main feature of the negative/positive dT/dP slope of ice VI and XV/XIX phase boundaries is common to all criteria. The relatively low transition temperature at 2.0 GPa might be caused by supercooling, which is a feature of first-order phase transition. In this study, the ratio, 2, was chosen as the criterion, because the criterion of 1.5 is occasionally too strict for the ratio obtained before the transition temperature; for example in the data of 1.6 GPa, the ratio is 1.42 and 1.37 at 122.5 and 125 K, respectively (Supplementary Fig. 15). If the criterion is too strict, the phase transition temperature would be overestimated. In addition, the ratio, 3.0, may underestimate the transition temperature considering such as the case of 1.7 and 2.0 GPa (Supplementary Fig. 15). In the phase diagrams, the provisional phase boundary between ice XV and XIX is denoted between two pressures where the transition temperature increases (decreases) with increasing

(decreasing) pressure. Also, transition temperatures of DCl-doped deuterated ice VI are shown. The D_2O samples show the compatible result with that obtained in H_2O ice, and also exhibit isotope effect in their phase transition temperatures, between which the difference is about 2 K. Such slight isotope effect has been reported in Ice I_h/XI and V/XIII phase transition (Koster et al. (2016)[1]), although we have no explanation for the isotope effect.

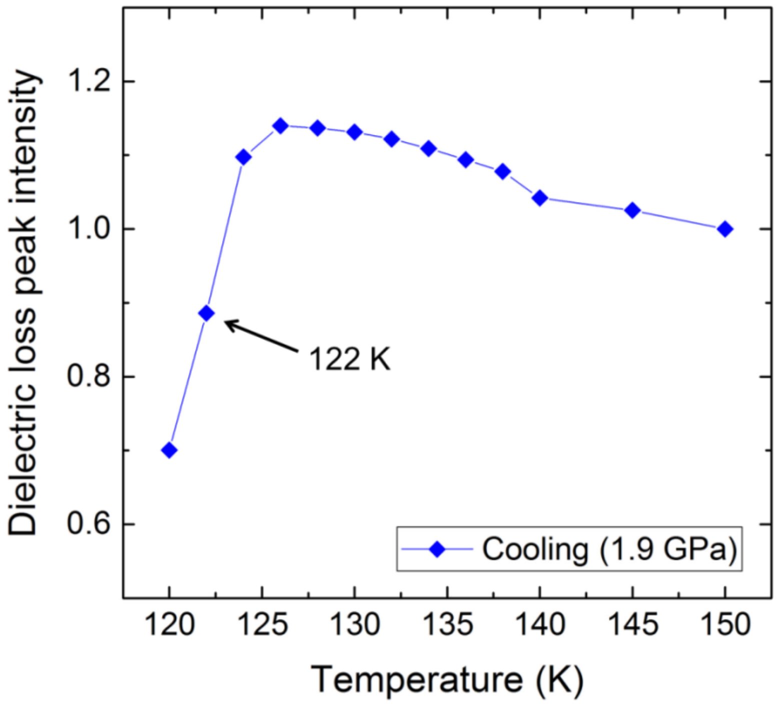

Supplementary Figure 13| Temperature dependence of dielectric loss peak intensity (DLPI) of HCl-doped ice VI and its hydrogen-ordered phase at 1.9 GPa upon cooling

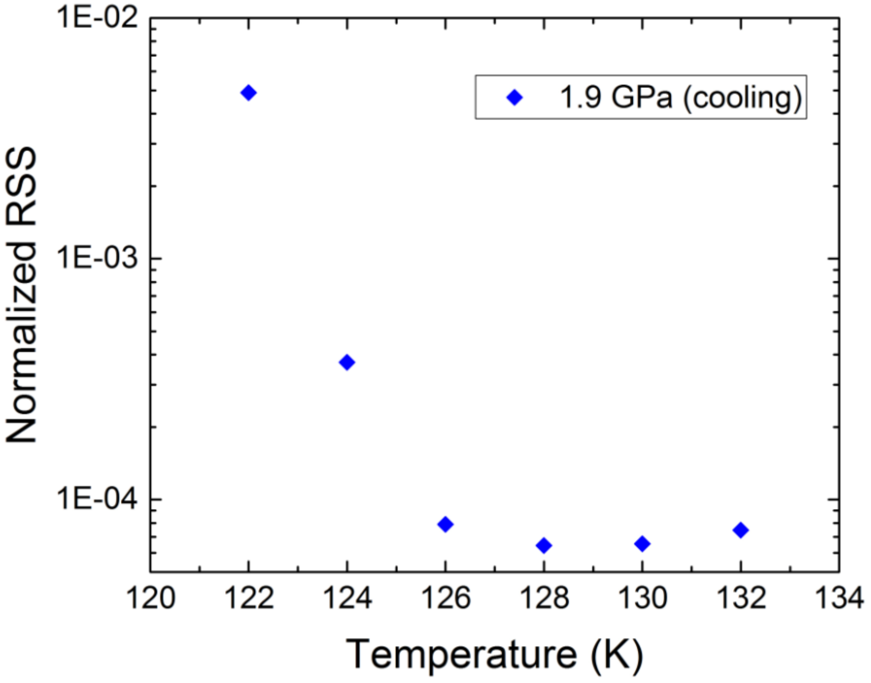

Supplementary Figure 14| Temperature dependence of normalized RSS in the cooling run at 1.9 GPa

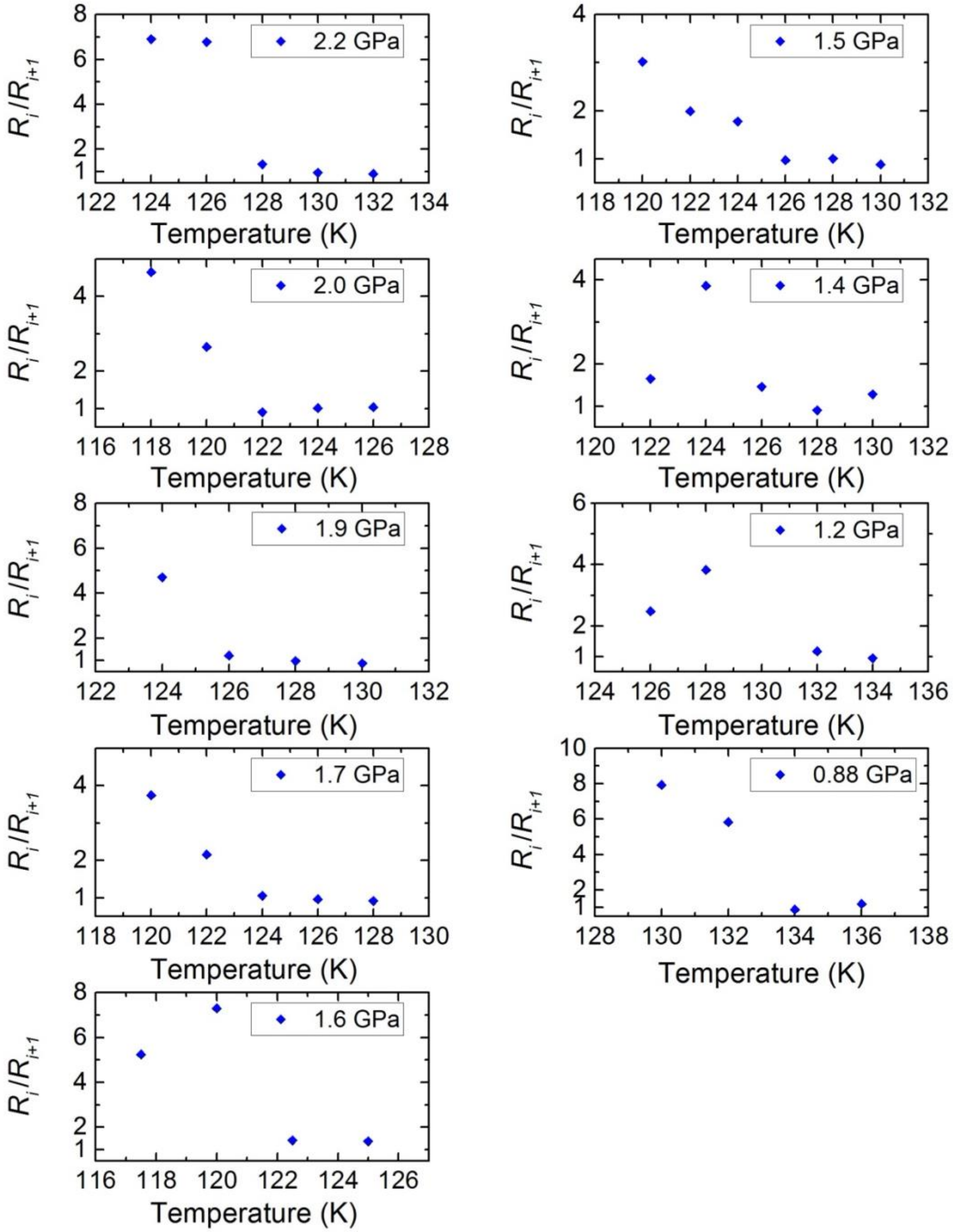

Supplementary Figure 15| Temperature dependence of the ratio R_i/R_{i+1} obtained in the pressure range 0.88–2.2 GPa upon cooling

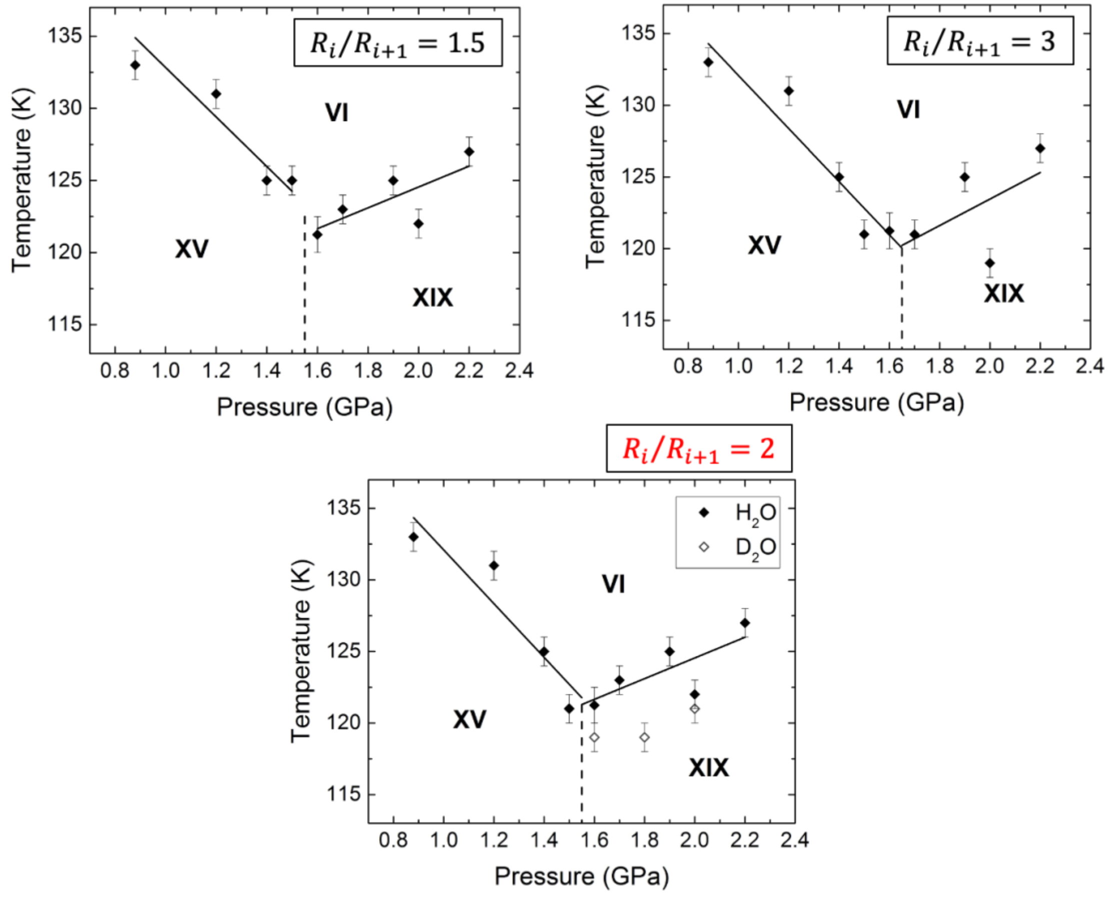

Supplementary Figure 16| Phase diagrams of ice VI and its hydrogen-ordered phases obtained from the several criteria

Supplementary Method 3 Details of the 18 candidates

Supplementary Figure 17 shows definitions of site labels of hydrogen atoms for the 18 structure models. Corresponded site occupancies of the hydrogen atom sites are represented by one or a few variables, denoted by Greek letters (Supplementary Table 5), which are actually fitting parameters for the Rietveld refinements using hydrogen site occupancies.

Supplementary Method 4 "Lower symmetry" space group

We only took account of 18 candidates of "higher symmetry" space groups for the structure analysis of ice XIX as mentioned in the main text. Considering two structure models, whose space groups are related by group-subgroup relationship, the subgroup structure model generally shows better agreement with the experimental data comparing with the structure model of its parent space group. Hence we set a criterion of "lower symmetry" space groups disregarded in this study based on a quantitative index, Group-Subgroup index. We consider a space group, represented by G. G is an infinite group due to its translation symmetry. Its translation operations make normal subgroup, H, of the space group, G. A coset of G by H, denoted by G/H, makes a quotient group. G/H is composed of finite elements and the number of elements is called the order of G/H, represented by $|G/H|$. For example, let us take $P1$ as an example space group of a crystal structure, S. When we choose a minimum unit cell of the S, $T(P1)$ is defined by all translation operations corresponding to the unit cell. Then, $|P1/T(P1)| = 1$ holds. Hereafter, we consider a case that S is the crystal structure of ice VI. In the case of the space group of ice VI, $P4_2/nmc$, $|P4_2/nmc/T(P4_2/nmc)|$ is 16. When the unit cell of ice VI is expanded to $\sqrt{2} \times \sqrt{2} \times 1$, the cell volume becomes two times larger. We represent corresponded translation operations as

$T_{\sqrt{2}\times\sqrt{2}\times 1}(P4_2/nmc)$. Then an order $|P4_2/nmc/T_{\sqrt{2}\times\sqrt{2}\times 1}(P4_2/nmc)|$ is 32. The Group-Subgroup index, *s*, is defined as follows:

$$s = \frac{\left|P4_2/nmc/T_{\sqrt{2}\times\sqrt{2}\times 1}(P4_2/nmc)\right|}{|H/T(H)|} = \frac{32}{|H/T(H)|},$$

where *H* is a subgroup of $P4_2/nmc$. For example in a case, $H = P\bar{1}$, Group-Subgroup index, *s*, is 16. Because, $P\bar{1}/T(P\bar{1})$ includes two elements; one is for trivial equivalent symmetry and the other one is about inversion symmetry. In this study, we ignored five space groups, *Pc*, $P2_1$, *P*2 and $P\bar{1}$ and *P*1, and this means that we only considered space groups, whose Group-Subgroup indexes are less than 8 from their sufficient refinement agreements for the neutron diffraction patterns. Space groups with indexes, 16 and 32, are *Pc*, $P2_1$, *P*2 and $P\bar{1}$ and *P*1, respectively. It is noted that subgroups of $P\bar{4}$ are *P*2 and *P*1 both of which belong to pyroelectric groups as well as *Pcc*2.

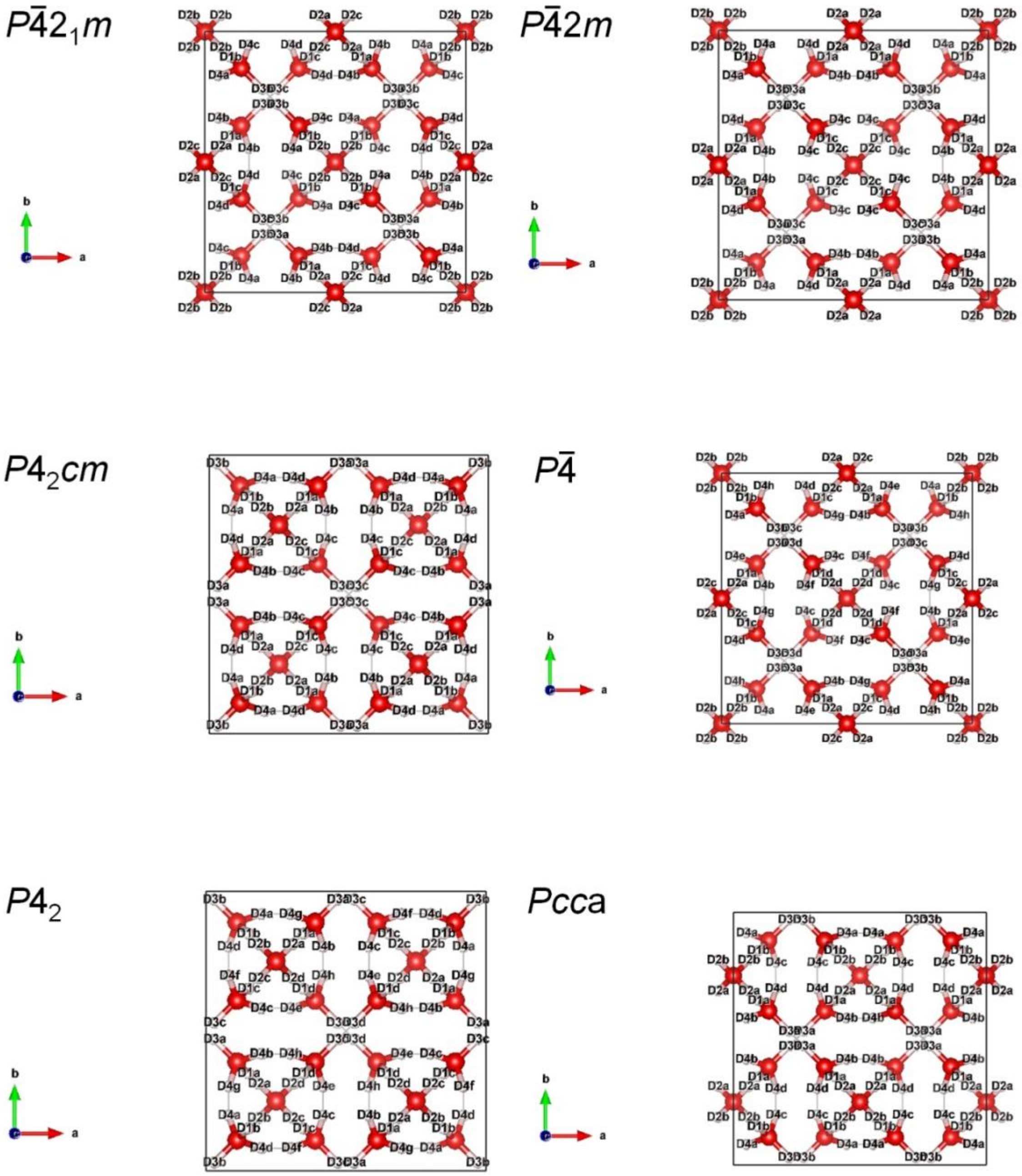

Supplementary Figure 17| Site labels of hydrogen atoms in the 18 structure models. Each corresponded space group is denoted left upper of its crystal structure. Black squares mean unit cells and each crystal axis is denoted in the left bottom.

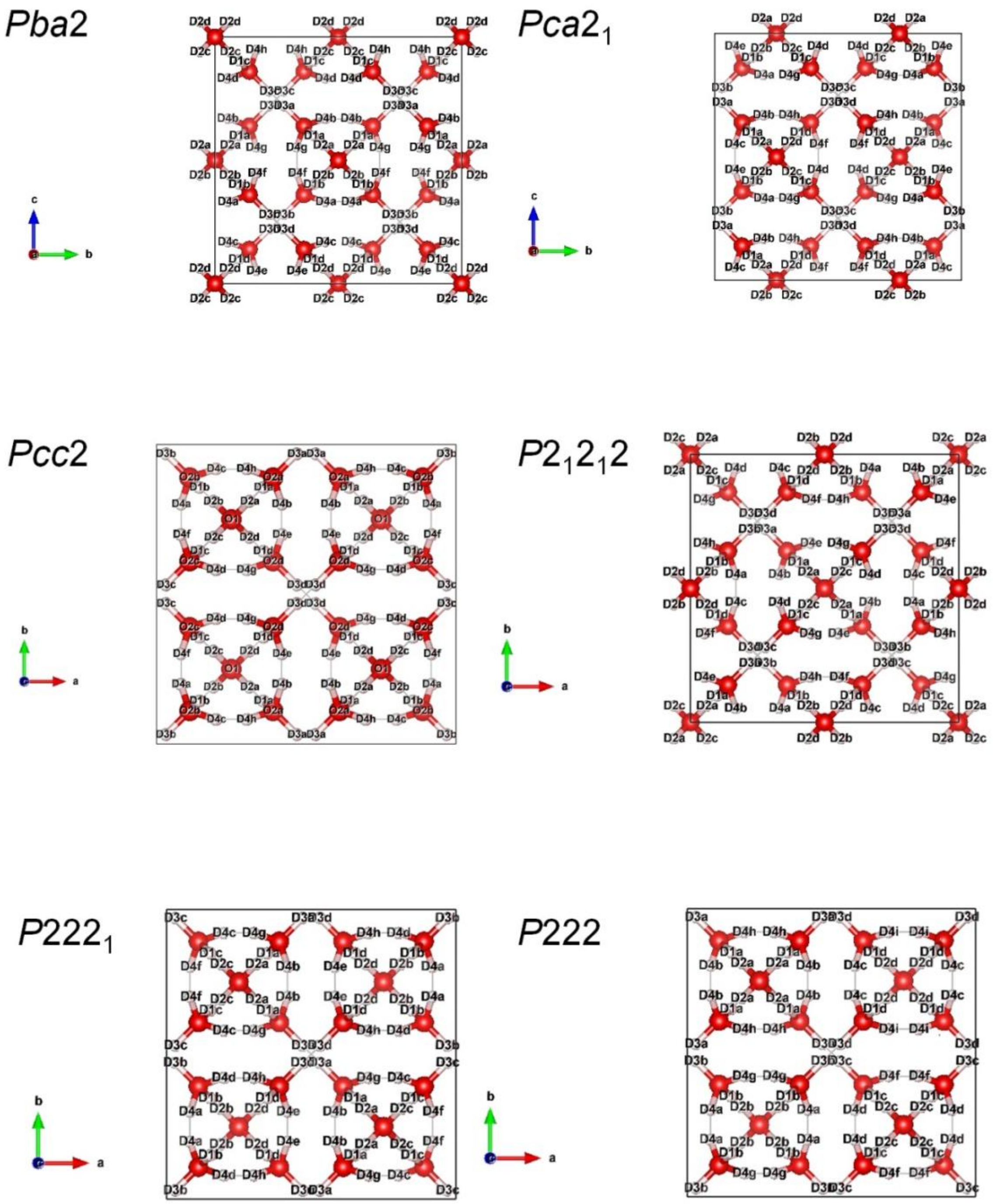

Supplementary Figure 17| (continuous) Site labels of hydrogen atoms in the 18 structure models. Each corresponded space group is denoted left upper of its crystal structure. Black squares mean unit cells and each crystal axis is denoted in the left bottom.

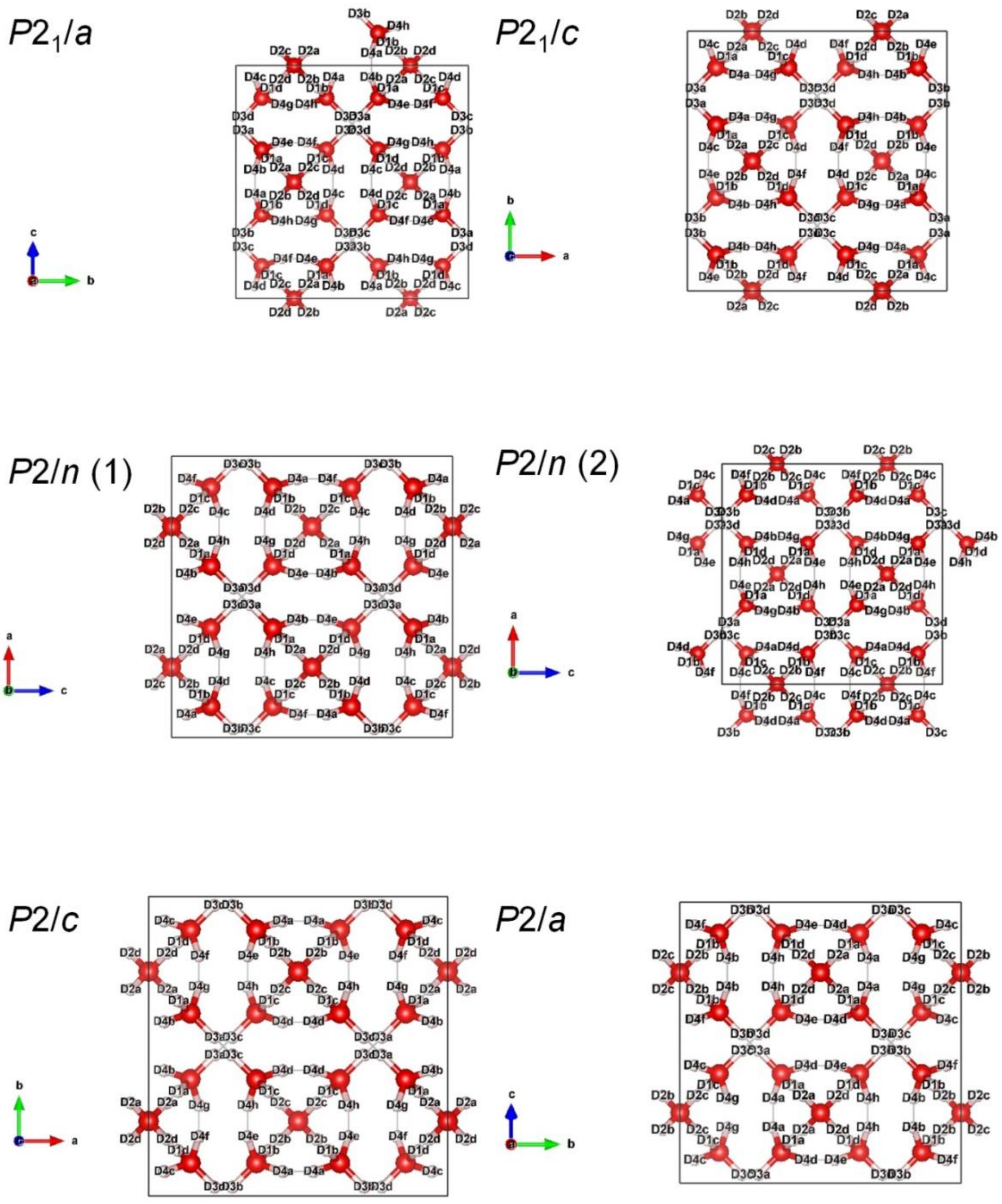

Supplementary Figure 17| (continuous) Site labels of hydrogen atoms in the 18 structure models. Each corresponded space group is denoted left upper of its crystal structure. Black squares mean

unit cells and each crystal axis is denoted in the left bottom. *P*2/*n* has two structure models, denoted by (1) and (2), depending on their geometric elements.

Supplementary Table 5| Representations of hydrogen site occupancies for the 18 structure models

#	Space group	Site occupancy of each hydrogen atom site
1	$P\bar{4}2_1m$	D1a : 1 - α, D1b : 0.5, D1c : α,<br>D2a : α, D2b : 0.5, D2c : 1 - α,<br>D3a : β, D3b : 0.5, D3c : 1 - β,<br>D4a : γ, D4b : 0.5 + (α - β)/2,<br>D4c : 1 - γ, D4d : 0.5 - (α - β)/2
2	$P\bar{4}2m$	D1a, D1b, D1c : 0.5, D2a, D2b, D2c : 0.5,<br>D3a : 0.5, D3b : α, D3c : 1 - α,<br>D4a, D4b, D4c, D4d : 0.5
3	$P4_2cm$	D1a : 1 - α, D1b : 1 - β, D1c : -1 + 2α + β,<br>D2a : α, D2b : β, D2c : 2 - 2α - β,<br>D3a, D3b, D3c : 0.5,<br>D4a : 0.25 + β/2, D4b : -0.25 + α + β/2,<br>D4c : 1.25 - α - β/2, D4d : 0.75 - β/2
4	$P\bar{4}$	D1a : 1 - α, D1c : α, D1b, D1d : 0.5,<br>D2a : α, D2c : 1 - α, D2b, D2d : 0.5,<br>D3a : β, D3c : 1 - β, D3b, D3d : 0.5,<br>D4a : γ, D4h : 1 - γ, D4c : δ,<br>D4f : 1 - δ, D4b : ϵ, D4g : 1 - ϵ,<br>D4d : -α + β + ϵ, D4e : 1 + α - β - ϵ
5	$P4_2$	D1a : 1 - α, D1b : 1 - β, D1c : 1 - γ, D1d : -1 + α + β + γ,<br>D2a : α, D2b : β, D2c :γ, D2d : 2 - α - β - γ,<br>D3a, D3b, D3c, D3d : 0.5,<br>D4a : δ, D4d : 0.5 + β - δ D4f : 0.5 - β + δ,<br>D4c : β + γ - δ, D4e : 1 - β - γ + δ, D4g : 1 - δ,<br>D4b : -0.5 + α + δ, D4h : 1.5 - α - δ
6	$Pcca$	D1a : 1 - α, D1b : α, D2a : α, D2b : 1 - α,<br>D3a, D3b : 0.5,<br>D4a, D4b : 0.5, D4c : 1 - α, D4d : α
7	$Pba2$	D1a : 1 - α, D1b : α, D1c : 1 - β, D1d : β,<br>D2a : α, D2b : 1 - α, D2c : β, D2d : 1 - β,<br>D3a, D3d : γ, D3b, D3c : 1 - γ,<br>D4a, D4b, D4c, D4d : 0.5,<br>D4e : 1.5 - β - γ, D4h : -0.5 + β - γ,<br>D4f : 0.5 - α + γ, D4g : 0.5 + α - γ
8	$Pca2_1$	D1a : 1 - α, D1b : 1 - β, D1c : 1 - γ, D1d : -1 + α + β + γ,<br>D2a : α, D2b : β, D2c : γ, D2d : 2 - α - β - γ,<br>D3a : δ, D3b : 1 - δ, D3c : ϵ, D3d : 1 - ϵ,<br>D4a : ζ, D4g : 1 - ζ, D4e : β + δ - ζ,<br>D4c : 1 - β - δ + ζ, D4b : α + β - ζ, D4h : 1 - α - β + ζ,<br>D4f : 1 - γ + ϵ - ζ, D4d : γ - ϵ + ζ

Supplementary Table 5| (continuous) Representations of hydrogen site occupancies for the 18 structure models

9	$Pcc2$	D1a : 1 - α, D1b : 1 - β, D1c : 1 - γ, D1d : -1 + α + β + γ,<br>D2a : α, D2b : β, D2c : γ, D2d : 2 - α - β - γ,<br>D3a, D3b, D3c, D3d : 0.5,<br>D4a : δ, D4b : α + β - δ, D4c : 0.5 + β - δ,<br>D4d : -0.5 + γ + δ, D4e : 1 - α - β + δ, D4f : 1 - δ,<br>D4g : 1.5 - γ - δ, D4h : 0.5 - β + δ
10	$P2_12_12$	D1a : 1 - α, D1b : 1 - β, D1c : α, D1d : β,<br>D2a : α, D2b : β, D2c : 1 - α, D2d : 1 - β,<br>D3a : γ, D3b : δ, D3c : 1 - γ, D3d : 1 - δ,<br>D4a : ϵ, D4c : 1 - ϵ, D4h : 1 + β - δ - ϵ,<br>D4f : -β + δ + ϵ, D4b : ζ, D4d : 1 - ζ,<br>D4e : 1 + α - γ - ζ, D4g : -α + γ + ζ
11	$P222_1$	D1a : 1 - α, D1b : 1 - β, D1c : α, D1d : β,<br>D2a : α, D2b : β, D2c : 1 - α, D2d : 1 - β,<br>D3a, D3b, D3c, D3d : 0.5,<br>D4a, D4b, D4e, D4f : 0.5,<br>D4c : 1 - α, D4g : α, D4d : β, D4h : 1 - β
12	$P222$	all 0.5
13	$P2_1/a$	D1a : 1 - α, D1b : 1 - β, D1c : 1 - γ, D1d : -1 + α + β + γ,<br>D2a : α, D2b : β, D2c : γ, D2d : 2 - α - β - γ,<br>D3a : δ, D3c : 1 - δ, D3b : ϵ, D3d : 1 - ϵ,<br>D4a : ζ, D4b : 1 - ζ, D4e : α - δ + ζ,<br>D4f : 1 - α + δ - ζ, D4d : -1 + α + γ + ζ, D4c : 2 - α - γ - ζ,<br>D4g : -β + ϵ + ζ, D4h : 1 + β - ϵ - ζ
14	$P2_1/c$	D1a : 1 - α, D1b : 1 - β, D1c : 1 - γ, D1d : -1 + α + β + γ,<br>D2a : α, D2b : β, D2c : γ, D2d : 2 - α - β - γ,<br>D3a : δ, D3b : 1 - δ, D3c : ϵ, D3d : 1 - ϵ,<br>D4a : ζ, D4g : 1 - ζ, D4d : γ - ϵ + ζ,<br>D4f : 1 - γ + ϵ - ζ, D4h : 1 - α - β + ζ, D4b : α + β - ζ,<br>D4e : -α + δ + ζ, D4c : 1 + α - δ - ζ
15	$P2/n$	D1a : 1 - α, D1b : 1 - β, D1c : 1 - γ, D1d : -1 + α + β + γ,<br>D2a : α, D2b : β, D2c : γ, D2d : 2 - α - β - γ,<br>D3a, D3b, D3c, D3d : 0.5,<br>D4a : δ, D4f : 1 - δ, D4c : -0.5 + γ + δ,<br>D4h : 1.5 - γ - δ, D4b : -1 + α + γ + δ, D4e : 2 - α - γ - δ,<br>D4g : 0.5 - β + δ, D4d : 0.5 + β - δ
16	$P2/n$	D1a, D2d : 1 - α, D1b, D2c : 1 - β,<br>D2a, D1d : α, D2b, D1c : β,<br>D3a, D3c : γ, D3b, D3d : 1 - γ,<br>D4a : δ, D4d : 1 - δ, D4f : -1 + β + γ + δ,<br>D4c : 2 - β - γ - δ, D4b : ϵ, D4g : 1 - ϵ,<br>D4e : α - γ + ϵ, D4h : 1 - α + γ - ϵ

Supplementary Table 5| (continuous) Representations of hydrogen site occupancies for the 18 structure models

17	$P2/c$	D1a, D2d : 1 - α, D1b, D2c : 1 - β,<br>D2a, D1d : α, D2b, D1c : β,<br>D3a, D3b : γ, D3c, D3d : 1 - γ,<br>D4a, D4b, D4c, D4d : 0.5,<br>D4e : 0.5 + β - γ, D4h : 0.5 - β + γ,<br>D4f : 0.5 - α + γ, D4g : 0.5 + α - γ
18	$P2/a$	D1a, D2d : 1 - α, D1b, D2c : 1 - β,<br>D2a, D1d : α, D2b, D1c : β,<br>D3a, D3c : γ, D3b, D3d : 1 - γ,<br>D4a, D4b, D4g, D4h : 0.5,<br>D4c : 1.5 - β - γ, D4f : -0.5 + β + γ,<br>D4d : 0.5 + α - γ, D4e : 0.5 - α + γ

Supplementary References

1. Köster, K. W. et al. Doping-enhanced dipolar dynamics in ice V as a precursor of hydrogen ordering in ice XIII. Phys. Rev. B **94**, 184306 (2016).
2. Kawada, S. Development of a new relaxational process having shortened relaxation time and phase transition in KOH-doped ice single crystal. J. Phys. Soc. Jpn. **58**, 295-300 (1989).
3. Johari, G. P., Lavergne, A. & Whalley, E. Dielectric properties of ice VII and ice VIII and the phase boundary between ice VI and VII. J. Chem. Phys. **61**, 4292-4300 (1974).
